# Unravelling the Adaptation Mechanisms to High Pressure in Proteins

**DOI:** 10.3390/ijms23158469

**Published:** 2022-07-30

**Authors:** Antonino Caliò, Cécile Dubois, Stéphane Fontanay, Michael Marek Koza, François Hoh, Christian Roumestand, Philippe Oger, Judith Peters

**Affiliations:** 1 Université de Lyon, UCBL, INSA Lyon, CNRS, MAP UMR 5240, 69621 Villeurbanne, France; antonino.calio@insa-lyon.fr (A.C.); stephane.fontanay@insa-lyon.fr (S.F.); 2 CBS, INSERM U1054, CNRS UMR 5048, Université de Montpellier, 34090 Montpellier, France; ceciledubois98@gmail.com (C.D.); hoh@cbs.cnrs.fr (F.H.); roume@cbs.cnrs.fr (C.R.); 3 Institut Laue Langevin, 38042 Grenoble, France; koza@ill.fr; 4 Université Grenoble Alpes, CNRS, LiPhy, 38400 Grenoble, France; 5 Institut Universitaire de France, 75231 Paris, France

**Keywords:** high pressure adaptation, protein dynamics, origins of life, neutron scattering

## Abstract

Life is thought to have appeared in the depth of the sea under high hydrostatic pressure. Nowadays, it is known that the deep biosphere hosts a myriad of life forms thriving under high-pressure conditions. However, the evolutionary mechanisms leading to their adaptation are still not known. Here, we show the molecular bases of these mechanisms through a joint structural and dynamical study of two orthologous proteins. We observed that pressure adaptation involves the decoupling of protein–water dynamics and the elimination of cavities in the protein core. This is achieved by rearranging the charged residues on the protein surface and using bulkier hydrophobic residues in the core. These findings will be the starting point in the search for a complete genomic model explaining high-pressure adaptation.

## 1. Introduction

According to one of the most credited hypotheses on the origins of life, it appeared in the deep sea [1], protected from the deleterious radiation of the young sun, but close to energy sources (e.g., hydrothermal vents) that could sustain relevant chemical reactions [2]. Therefore, it would have appeared under high hydrostatic pressure (HHP) conditions. Modern HHP-adapted organisms (piezophiles) display pressure-dependent physiology; however, deciphering their adaptation to HHP is a very challenging task because it is often concomitant with other environmental adaptations [3]. Indeed, they usually thrive in very cold [4], i.e., the deep ocean, or very hot environments [5], i.e., hydrothermal vents. The first demonstration of proteome structural adaptation in piezophiles originated from comparative whole-cell studies between two nearly isogenic piezophilic and piezosensitive microorganisms, namely *Thermococcus barophilus* and *Thermococcus kodakarensis*, which share identical growth characteristics, except for HHP adaptation. Their proteomes exhibit different dynamical properties, together with a remarkable difference in the response to HHP of the hydration water [6]. In contrast to accepted models, proteins of the piezosensitive microorganism appear less sensitive to increasing HHP, while those of the piezophile are more flexible, and undergo pressure-dependent rearrangements at a pressure value close to the optimum of the organism [6,7]. Above this threshold, the piezophile proteome becomes pressure-insensitive [6,7,8,9]. Thus, unexpectedly, the adaptation to HHP in piezophiles seems to imply that the cell’s proteome is both more sensitive and more resistant to HHP [6]. These dynamical characteristics can also be preserved by piezophile cells under low-pressure stress through the accumulation of organic osmolytes [10]. Interestingly, a similar insensitivity to HHP has been observed for concentrated protein solutions in the presence of organic osmolytes [11]. Another peculiarity of piezophiles is the response of their proteome’s hydration shell to HHP: its size is reduced and water appears less mobile [6]. It is thus probable that structural adaptation in proteins of piezophiles affects amino acids at the water–protein interface. Therefore, two different processes seem to be responsible for HHP adaptation: (i) a structural (i.e., genomic) adaptation, modifying protein sequences to alter their dynamics, and/or (ii) the modulation of protein–water interaction. To date, all attempts to identify the structural signature of HHP adaptation at the genome level have failed, likely because it only involves amino acids that interact with the hydration water or take part in the formation of internal cavities, which can be greatly destabilized by HHP [12]. If the macroscopic thermodynamics of proteins under pressure is quite well established [12,13,14], its influence on their microscopical properties and their dynamics is still a debated subject [15,16,17,18,19,20,21]. Many different, and often contrasting, contributions govern the structural and dynamical stability of proteins with respect to HHP [14], such as the presence of solvent inaccessible cavities [12], electrostriction [22] and the pressure dependence of the hydrophobic effect [23]. Concerning the fast dynamics, there is evidence that pressure tends to slow it down and inhibit conformational changes that require large amplitude motions [15,21]. To investigate protein HHP adaptation, Elastic Incoherent Neutron Scattering (EINS) and Quasi-Elastic Neutron Scattering (QENS) have been employed to study the dynamics of two orthologous proteins from *T. barophilus* (Tba) and *T. kodakarensis* (Tko), which only differ by their optimal growth pressure. Our data show profound differences both in their dynamics and in their interaction with the surrounding water layer, giving the first hints about the molecular mechanisms involved in HHP adaptation.

## 2. Results

### 2.1. Crystal Structures and Molecular Dynamics Simulations

**Figure 1:**
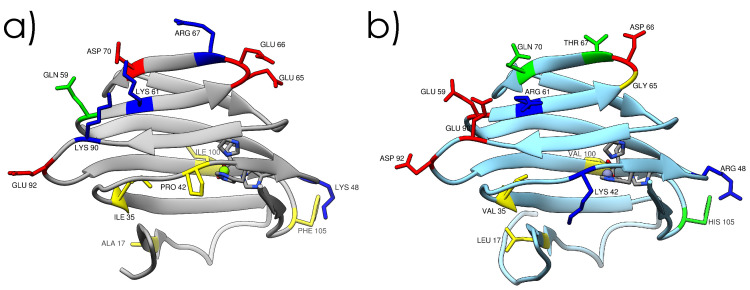
Crystal structures of Tba PMI (**a**) and Tko PMI (**b**) with labelled substituted residues for comparison. Residues are color-coded for polarity (red for acidic, blue for basic, green for polar, and yellow for hydrophobic), and the active site is also shown (details in Figure S7).

**Figure 2:**
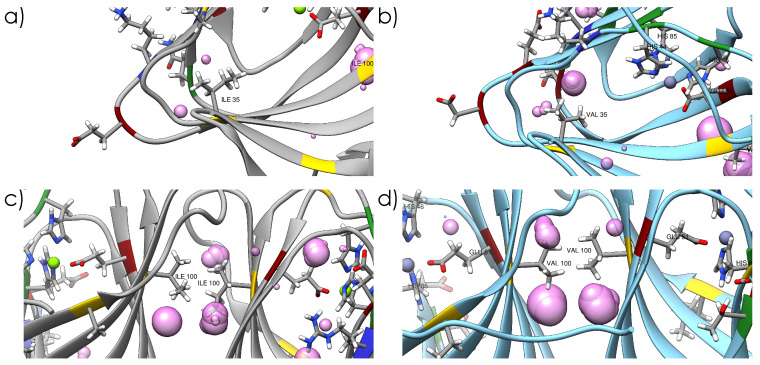
Internal cavities in the two proteins located near Ile-Val substitutions in the protein core (**a**,**b**) and at the dimer interface (**c**,**d**) after the relaxation of the crystal structures by MD at 310 K and 1 bar.

Genes TERMP_00744 and TK_0503, coding for a *Phosphomannose isomerase* (PMI), were cloned into the pET-16b over-expression vector [24] for Neutron Scattering experiments, and in the pT7-7 vector [25] for crystallization. The two PMIs have been overexpressed in *E. coli* (BL21 (DE3) pLysS) and purified by heat-treating the cell lysate and by size-exclusion chromatography (Figure S3, see Section 4 for further details). X-ray crystallography was performed to obtain the structures of the proteins (Figure 1 and Figure S5). Both present very similar features: their structure is dominated by β-sheet contributions with turns and disordered regions connecting them, forming a jelly-roll barrel structure, and both present a dimeric quaternary structure. The active site is located in a pocket inside the barrel (Figure S6) and is conserved between the two proteins (His44, His46, Glu51 and His85). Interestingly, the catalytic metal ion coordinated by these four residues is found to be magnesium in Tba PMI and zinc in Tko PMI; however, the structure’s resolution does not allow for making a definitive assignment, as the Van der Waals radius of both ions is smaller than the resolution. The two orthologs differ at 16 positions (Figure S1), and most of the substitutions are located at the protein–water interface and involve mainly polar and charged residues (Figure 1), with the notable exceptions of I35V, located in the hydrophobic core of the protein (Figure 2a,b), and I100V, located at the monomer–monomer interface (Figure 2c,d). The crystal structures have been subsequently relaxed and equilibrated in solution through Molecular Dynamics (MD) simulations, and the internal cavities have been revealed (Figure 2). This already shows the impact that these two seemingly conservative substitutions have on the internal packing of the two proteins. It must be noted that the length of the trajectories may not be sufficient to fully relax the structures, but it is compatible with the time-scale of the neutron scattering experiments and would thus reasonably reproduce the structural and dynamical properties probed by these techniques. Moreover, it must be noted that the cavities are probed on average representative structures; therefore, their fluctuations in time are neglected.

### 2.2. Elastic Incoherent Neutron Scattering (EINS)

**Figure 3:**
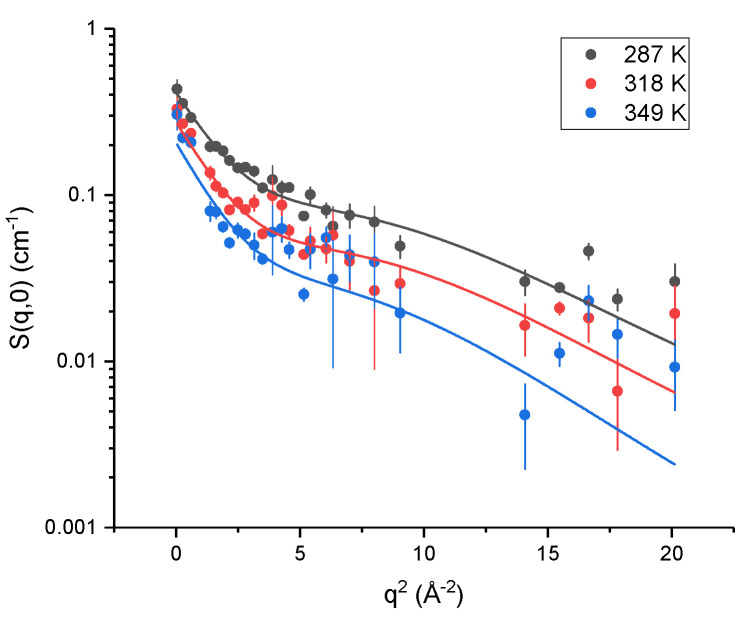
Scattering curves for Tba PMI at 150 bar and at some representative temperatures, with the corresponding two-state model fits. The decrease of the elastic intensity with temperature is expected, as more motions enter the time window of the experiment and the involved atoms scatter neutrons inelastically.

EINS was used to access the motions of the two proteins [26,27] in solution, and to probe their response to HHP, while considering their structural differences, to shed light on their adaptation strategies. Incoherent neutron scattering probes the single-particle self-correlation function [28] and, in protein samples, the signal is dominated by hydrogen atoms, thanks to their very large incoherent cross-section [29]. In the case of elastic scattering, there is no energy exchange between the incident neutrons and the sample, meaning that, in the time domain, the correlation function is probed in the long-time limit. Given that the instrument has a finite energy resolution (8 μeV for IN13, see Section 4), this time limit is not at infinity, but it defines the *time window* of the instrument (∼100 ps for IN13). Figure 3 shows the scattering curves for Tba PMI at three representative temperatures. As expected, the scattering intensity shows a general decrease with temperature, consistent with the activation of anharmonic motions [30]. Some contributions from global diffusion of the protein are likely present given the low concentration of the samples. Nonetheless, due to the very close similarity in the primary sequence and the almost identical molecular weight of the two proteins, it is reasonable to assume that these contributions would similarly affect the measured signal for both samples. Hence, the observed differences can be ascribed to the distinct internal dynamics of the two proteins.

**Figure 4:**
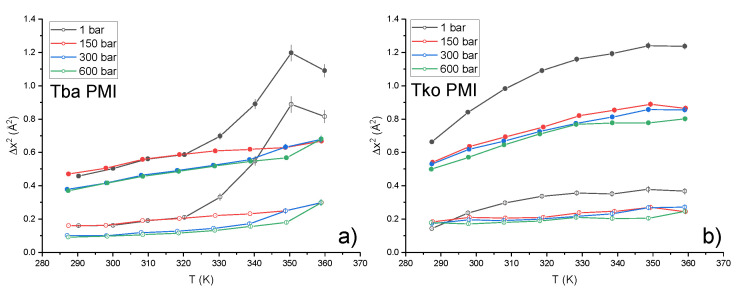
Total MSD (full circles) and MSD into the single wells ($\Delta x_{0}^{2}$, open circles) for Tba PMI (**a**) and Tko PMI (**b**), lines are a guide to the eye. The absolute value of the MSD reports on the amplitude of hydrogen atoms motion, while the slope of the curve as a function of temperature indicates how much energy is necessary to increase said amplitude, i.e., the *resilience* of the protein [32].

Data have been interpreted in the framework of a two-state model [31], which assumes two harmonic potential wells with an associated Mean Square Displacement (MSD) $\Delta x_{0}^{2}$, separated by a distance *d* and a free energy ΔG=ΔH−TΔS (see Methods). The temperature independence of *d* (Figure S10), ΔH and ΔS (consistently with the assumed Arrhenius behaviour of the two wells’ populations) allowed the employment of a global fitting procedure at each pressure point, in which the only temperature-dependent parameter was $\Delta x_{0}^{2}$. This granted the minimization of the number of free fitting parameters and greatly improved the quality and stability of the fittings. Figure 4 shows, for both samples, the single-well MSD, $\Delta x_{0}^{2}$, and the total MSD, $\Delta x_{tot}^{2}=\Delta x_{0}^{2}+\frac{p_{1}p_{2}}{3}d^{2}$, which takes into account the jump distance between the two wells and their populations. The absolute values of $\Delta x_{tot}^{2}$, which report on the amplitude of the internal motions, appear very similar for both samples around 350 K, indicating that the proteins display a similar degree of flexibility in proximity of the optimum growth temperature for both organisms. However, the two samples show a clearly different temperature dependence of the MSD, i.e., the slope of the curve which is inversely proportional to the protein’s resilience [32,33]: while Tko PMI displays a smooth increase, a change of slope in Tba PMI at around 320K and 1 bar evidences the lower resilience, i.e., higher *softness*, of the protein. This transition is not present at higher pressures, and a linear temperature dependence of the MSD is observed.

**Figure 5:**
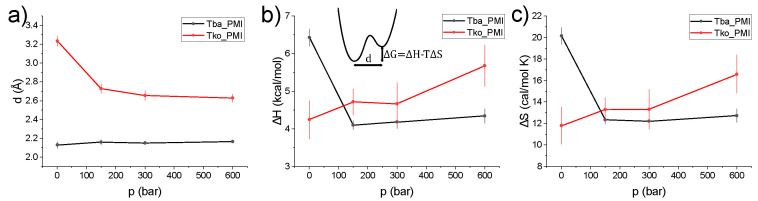
Temperature-independent parameters extracted from the two-state model as a function of pressure for Tba PMI (black symbols) and Tko PMI (red symbols): distance between the two wells (*d*, (**a**)), enthalpy (ΔH, (**b**)) and entropy (ΔS, (**c**)) difference, lines are a guide to the eye. (**b**) also contains a pictorial representation of the model showing how the different parameters influence the shape of a proteins’ energy landscape.

These results are in line with those from whole-cell studies on the same two organisms, which demonstrated the higher flexibility of the proteome of *T. barophilus* [6,7,34] and the existence of pressure-induced structural rearrangements [7,34] in this strain. For *T. barophilus*, the transition occurs smoothly between 1 and 300 bar, close to the optimal growth pressure for the organism, i.e., 400 bar [35]. In *T. kodakarensis*, the transition takes place at a much lower pressure range as shown by the sharp decrease of the MSD from 1 to 150 bar. Further striking differences between the two proteins can be found by looking at the pressure dependence of the other parameters extracted from the two-state model fitting (Figure 5). In particular, the distance between the wells, *d* (panel a), is essentially pressure-independent for Tba PMI, while a sizeable decrease is detected for Tko PMI. This behaviour has already been observed in the model protein myoglobin [21] and has been explained in terms of an increased roughness of the protein energy landscape, arising from the difficulty of the protein to explore the conformational substates characterized by bigger volume differences, in agreement with Le Châtelier’s principle [36]. In contrast, the energy landscape of Tba PMI appears to be extremely stable with respect to pressure application, resembling what Shrestha et al. [20] found for the *T. thioreducens* IPPase. However, in that case, the comparison with the piezosensitive counterpart was considerably less significant, as hen egg-white lysozyme is not related to the IPPase. The stability of Tba PMI is confirmed by the behaviour of the thermodynamic parameters ΔH (Figure 5b) and ΔS (Figure 5c), as they both decrease sharply from 1 to 150 bar and then become pressure-independent, while they both increase for Tko PMI. A decrease in the ΔS has been connected with decreased hydration [31] and, indeed, such a decrease in the hydration shell size has been detected in the piezophilic proteome [6]. This is consistent with the two proteins having different interactions with water. The exclusion of water from the hydration shell of Tba PMI appears to be the key to the dynamical stability of the protein under high pressure.

### 2.3. Quasi-Elastic Neutron Scattering (QENS)

QENS was used to extract detailed information on the fast dynamics of the protein in solution, probe the effect of HHP, and reveal how specific substitutions in the sequences could affect them, giving insight into the mechanism of pressure adaptation.

**Figure 6:**
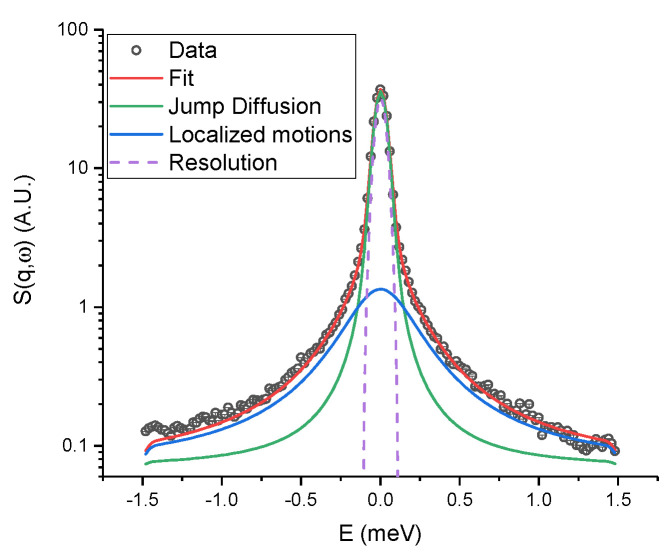
Fit example of Tba PMI at 1 bar and 286 K, at a *q* value of 1.14 Å^−1^. Black circles represent the corrected data, the two Lorentzian contributions owing to localized motions and jump–diffusion are shown respectively as blue and green solid lines, and the total fit is shown as a red solid line. The resolution function is also shown. Error bars are smaller than the symbol size.

**Figure 7:**
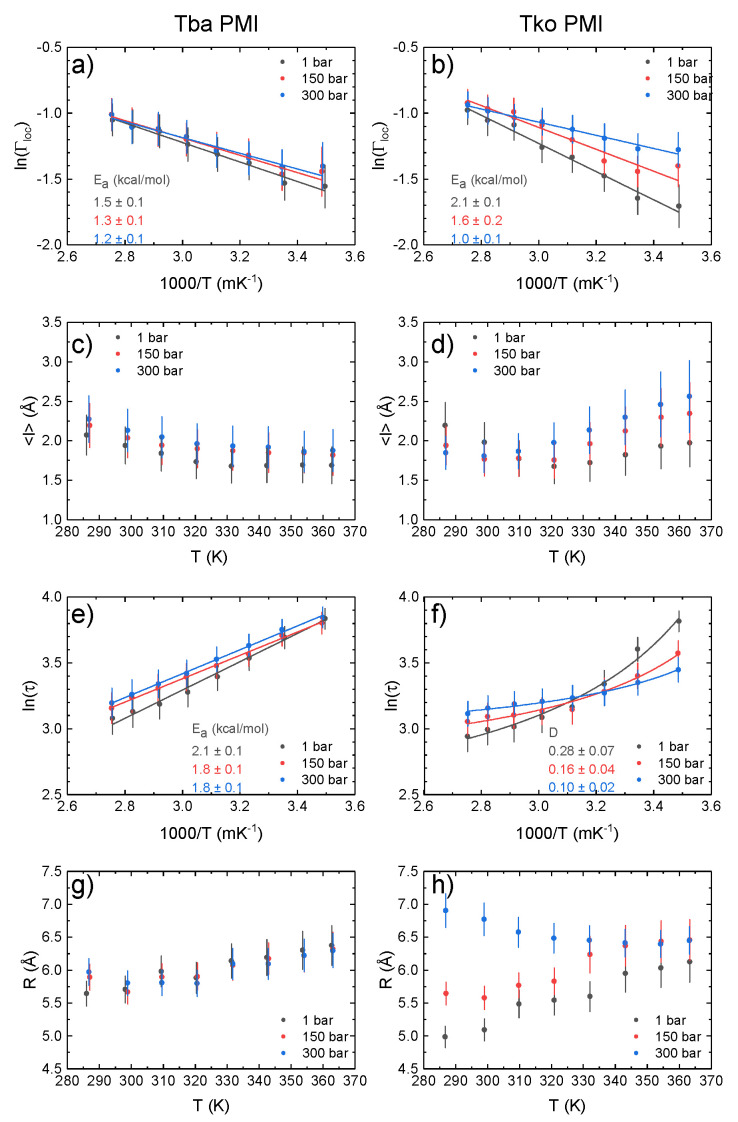
Natural logarithm of the broad component HWHM as a function of inverse temperature for Tba PMI (**a**) and Tko PMI (**b**) at all pressure values. Lines are linear fittings assuming an Arrhenius behaviour. Activation energy values are reported on the figure color-coded with the plots. Mean jump length from the Hall–Ross model as a function of temperature and pressure for Tba PMI (**c**) and Tko PMI (**d**). Natural logarithm of the residence time as a function of inverse temperature at all pressure values. Lines are fits to the Arrhenius law ((**e**), Tba PMI) or to the Vogel–Fulcher–Tamman law ((**f**), Tko PMI). Values for the activation energy (Tba PMI) and the fragility index (Tko PMI) are reported on the figure color-coded with the plots. Values of confinement radius *R* for Tba PMI (**g**) and Tko PMI (**h**).

As in EINS, the QENS signal is dominated by the incoherent contribution of hydrogen atoms, but in this case, a small energy transfer between the incident neutrons and the sample is allowed: it is thus possible to separate the contributions to the signal arising from different motions, while EINS gives an average picture. The analysis of the spectra gives access to localized and diffusional motions taking place on a specific time scale, which is ∼10 ps for IN5, and to characterize their geometry. Figure 6 shows a fit example, in which the two components we identified are highlighted: a broad and *q*-independent contribution due to fast localized motions, and a narrow contribution arising from confined jump–diffusion of protein residues. The logarithm of the HWHM of the broad component (Figure 7a,b) follows an Arrhenius behaviour ($\Gamma_{loc}\left(T\right)=\Gamma_{0}\exp\left(-E_{A}/RT\right)$, where $E_{A}$ is the activation energy, *R* is the gas constant and $\Gamma_{0}$ is the pre-exponential constant) at all pressure values for both samples. Tba PMI shows enhanced pressure stability compared to its piezosensitive counterpart concerning fast localized motions. The activation energy values suggest that the rotation of methyl groups is the dominant process from which this contribution arises (values ranging from 1.5 to 3.8 kcal/mol have been reported [37]). Moreover, the value of $E_{A}$ for methyl rotations has been shown to decrease in efficiently packed hydrophobic environments [37]. Hence, our data indicate that the extent of compression of the protein hydrophobic core is larger in Tko PMI than in Tba PMI, presumably due to the presence of bigger cavities in the former [12]. Concerning the narrow component, the extracted parameters are the mean jump length *<l>* and the residence time τ, which represents the mean time between two successive jumps.

**Figure 8:**
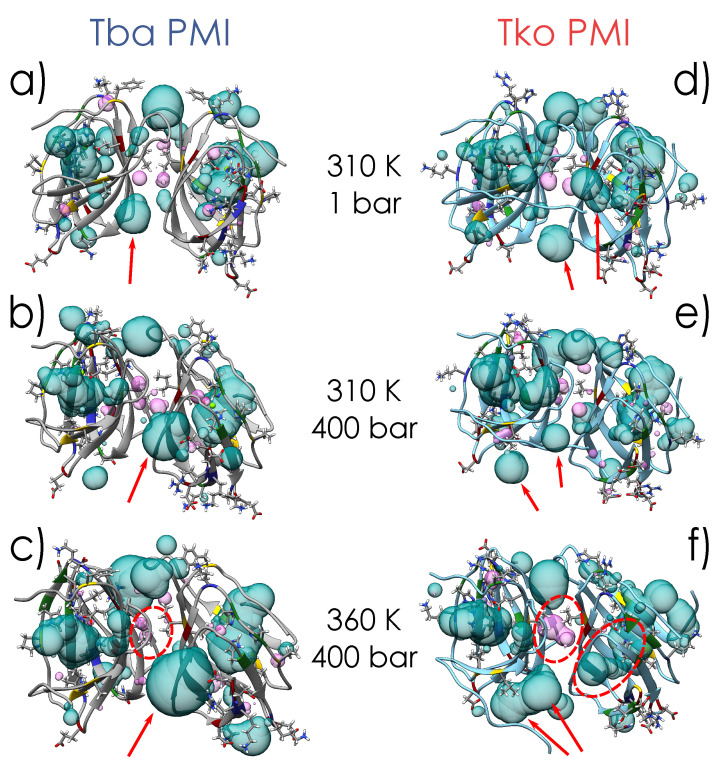
Representation of internal (magenta) and solvent-accessible (cyan) cavities for Tba PMI (**a**–**c**) and Tko PMI (**d**–**f**) at different T and P conditions. Structures are represented in cartoons, and substituted residues are evidenced in sticks (with the corresponding part of the ribbon coloured for residue type, blue for basic, red for acidic, green for polar and yellow for hydrophobic). Red arrows follow the evolution of some cavities, and the opposite behaviour of the internal cavities in the dimer interface of the two proteins is highlighted by red circles. Another red circle in (**f**) also shows how the ligand pocket in one monomer of Tko PMI has actually become a channel from side to side under extreme conditions.

The temperature dependence of *<l>* (Figure 7c,d) for Tba PMI is rather weak and does not change with pressure, testifying to the structural stability of the protein in the whole temperature and pressure range studied. For Tko PMI, this quantity shows a similar behaviour at 1 bar, where the protein is expected to be functional, while higher pressures seem to have a destabilizing effect. While it would appear straightforward to compare this quantity to the distance between the wells *d* derived from EINS data (Figure 5a), it must be stressed that the latter results from *all* the internal motions that are activated on the 100ps time scale, and thus gives an average representation of the protein’s energy landscape, while *<l>* refers to a particular motion, namely the jump–diffusion of side chains, and it relates to a different time scale. Furthermore, for the sake of comparison with other works, a *pseudo*-diffusion coefficient related to internal dynamics can be calculated according to $D_{pseudo}={<l>}^{2}/2\tau$ [38] (Figure S15). The difference in the temperature dependence of τ (Figure 7e,f) for the two proteins is remarkable: it follows the Arrhenius law ($\tau \left(T\right)=\tau_{0}\exp\left(E_{A}/RT\right)$, note the sign reversal compared to before, as τ=ℏ/Γ) for Tba PMI, while it follows the Vogel–Fulcher–Tamman (VFT) law ($\tau \left(T\right)=\tau_{0}\exp\left(\frac{DT_{0}}{T-T_{0}}\right)$, where 1/D is the fragility index, not to be confused with the aforementioned pseudo-diffusion coefficient $D_{pseudo}$, and $T_{0}$ is the Vogel temperature) for Tko PMI. The latter is typical of glass-forming systems [39,40,41], but it has also been observed in proteins and interpreted as the signature of protein–water coupled dynamics [42,43,44,45]. An Arrhenius behaviour arises from activated processes, and it is expected for jump–diffusion, while a VFT behaviour is usually connected with cooperative processes. The appearance of VFT behaviour on such a fast time-scale is intriguing, and it shows that side-chain relaxations are strongly coupled to hydration water dynamics in Tko PMI. This correlates with the higher ΔS in the EINS data and suggests that the decoupling of protein dynamics from its environment could be the key to pressure adaptation for Tba PMI. To push the analogy further, materials with a low fragility index exhibit a linear behaviour far from the Vogel Temperature $T_{0}$ which can be reasonably fitted with the Arrhenius law, and are referred to as *strong* glass-forming materials, while *fragile* materials show VFT behaviour in a considerably wider temperature range [46]. Thus, the difference in dynamical properties between Tba PMI and Tko PMI could be assimilated to that between strong and fragile glass-forming materials. It appears that Tko PMI’s dynamics are dominated by cooperative motions and that high pressure can destabilize them, as highlighted by the increase in fragility (i.e., decreasing *D*) with increasing pressure, while Tba PMI’s dynamics are dominated by pressure-insensitive activated processes. This difference is likely arising from the distinct amino-acidic composition of the two proteins, and it could explain the superior pressure stability of Tba PMI. The *Elastic Incoherent Structure Factor* (EISF) gives information about the geometry of the motions inside the resolution of the instrument, and has been analysed as described in the Methods section (Figure S16 and Figure S17). The behaviour of the confinement radius *R* extracted from the EISF (Figure 7g,h) highlights another remarkable difference between the two proteins. Tba PMI shows a weak temperature dependence of this parameter, compatible with thermal expansion, and again shows no pressure dependence. On the other hand, the temperature dependence of Tko PMI appears to be stronger, and a sizeable increase in the confinement radius is detected with increasing pressure. This result could appear counter-intuitive, but it can be rationalized by thinking of *R* as an average measure of the protein’s solvent-accessible cavities: higher pressure forces water into them, increasing their volume, while concomitantly decreasing the protein’s specific volume, in agreement with Le Châtelier’s principle. This volume increase in Tko PMI could also explain the enhanced mean jump length seen at high pressure (Figure 7d) and the stronger coupling with water displayed by the protein side-chains (Figure 5c and Figure 7f). Such assignment is substantiated by the results of MD simulations: an equivalent radius (i.e., the radius of a sphere with the same volume) has been calculated from the volume of the ligand pocket of both proteins simulated in different conditions (Figure S9). At low pressure and temperature, the found value is remarkably close to what is found by QENS, as the ligand pocket is the dominant solvent-accessible cavity in both proteins. However, the values deviate at high temperature and pressure, as QENS gives an average measure of *all* the cavities in the protein, and more cavities become of comparable size to the ligand pocket under extreme conditions. Nevertheless, MD values follow a very similar trend to the QENS values, and this can also be seen visually in Figure 8.

## 3. Discussion

**Figure 9:**
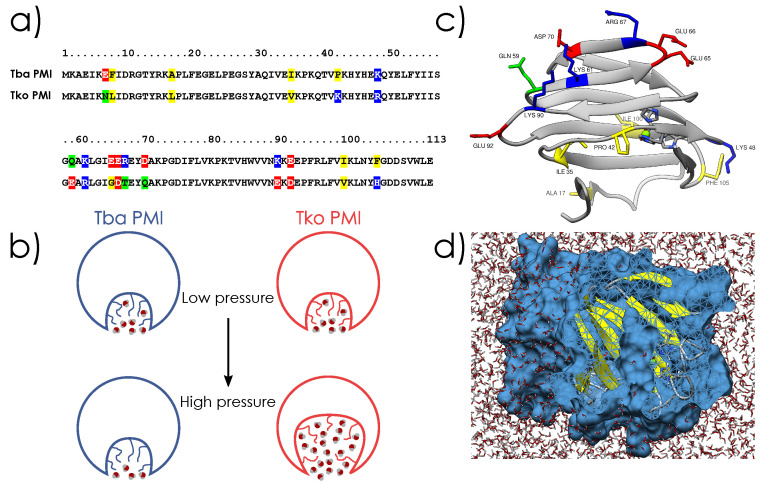
(**a**) sequence alignment of the two proteins, substitutions are highlighted (red for acidic, blue for basic, green for polar and yellow for hydrophobic residues). (**b**) schematic representation of the two proteins and effect of high pressure on them. (**c**) cartoon representation of Tba PMI, substitutions are represented in sticks with the same color-code as (**b**). (**d**) vertical cut on the surface representation of Tba PMI, highlighting the ligand pocket.

This study aims to compare proteins under positive selection originating from nearly isogenic organisms, differing only in their adaptation to pressure. Hence, for the first time, the different dynamical properties exhibited by the two proteins, as revealed by Neutron Scattering, and their structural properties, elucidated by X-ray crystallography and Molecular Dynamics simulations, can be correlated to decipher the adaptation to HHP regardless of other adaptative traits. The results show that the mechanisms by which Tba PMI counteracts the effect of HHP are: (i) the prevention of excessive amounts of water from penetrating the solvent-accessible cavities and limiting their destabilization, (ii) the inhibition of protein–water cooperative relaxations, and (iii) the reduction of internal (i.e., inaccessible to the solvent) cavity volume. This becomes of particular importance when considering the ligand pocket of the protein, i.e., the largest solvent-accessible cavity: its structure must be preserved under HHP to properly carry out the enzymatic reaction (Figure 9c) and to play its role in the metabolism of the organism.

Because of these results, it is possible to characterize the impact of each substitution in the two protein sequences by comparing their structures and dynamics. The substitutions will be identified with the first letter being the residue present in Tba PMI, and the second letter for that in Tko PMI. The two proteins differ at 16 positions and, as shown in the 3D representation of the protein (Figure 9a,b), all of the substituted residues present their side-chain exposed to the solvent, except for I35V and I100V. It is known that even very small volume changes in the interior of a protein (such as a single point mutation) can greatly affect its pressure stability, especially when this residue is located close to an internal cavity and the substitution is affecting its volume [12]. To this extent, the I35V substitution appears to be key in the stabilization of the hydrophobic core (Figure 2a,b), with the added importance of being close to the ligand pocket, limiting its deformation under extreme conditions (Figure 8). The active site is very conserved except for K48R, which is not affecting charge or volume, and P42K, where the proline residue might soften the rigid β-strand structure and render it more resilient to pressure changes in Tba PMI. In the dimer-forming region, the I100V substitution optimizes the contact between the two monomers and decreases internal cavity volume (Figure 2c,d), stabilizing Tba PMI’s quaternary structure under extreme conditions and seemingly maintaining optimal functionality (Figure 8). The remaining substitutions (E7N, Q59E, K61R, E65G, E66D, R67T, D70Q, K90E, E92D, F105H) are located at the protein–water interface and are likely involved in the modulation of the protein’s surface charge distribution, affecting its coupling with hydration water. This is visible when calculating the electrostatic potential on the surface of both proteins (Figure S8): Tba PMI shows a higher positive potential than its piezosensitive counterpart around the entrance of the ligand pocket, a higher negative potential in the middle region, and less uncharged regions in general. This excess charge could in turn increase the orientational constraints on the water molecules in the first hydration shell, effectively shielding the protein. Furthermore, the charge reorganization on Tba PMI’s surface promotes the formation of salt bridges (e.g., Lys61 with Asp70), applying different constraints on the protein structure and optimizing its response to high pressure, while also hindering the interaction that those residues could have with water molecules. Two more substitutions are found in F8L and A17L; however, they do not appear to be involved in any particular mechanism, and will thus be considered random conservative mutations.

The overall effect of these substitutions on Tba PMI is to efficiently maintain the protein structure, especially around the ligand pocket. On the contrary, at 360 K and 400 bar, Tko PMI’s ligand pocket substantially becomes a channel (Figure 8f) spanning from one side to the other of the monomer, and we speculate that protein function would be at best severely hindered in these conditions. Hence, the amino-acid substitutions between the two PMIs allow for drawing a first adaptation pattern related to HHP. This is characterized by a decrease in polar residues in favour of charged ones, particularly glutamate and lysine, in the piezophilic protein. Moreover, precise substitutions involving isoleucine instead of valine enable the piezophilic protein to tailor its occupation of void volume both in its core and at the dimerization interface, enhancing its pressure stability. However, the relative contributions of the different amino-acid substitutions identified in this work need to be confirmed by further studies using direct methods (multidimensional NMR spectroscopy, site-directed mutagenesis), and eventually other piezophilic proteins.

## 4. Materials and Methods

### 4.1. Protein Expression and Purification

Recombinant Phosphomannose Isomerases from *T. barophilus* and *T. kodakarensis* have been produced by cloning synthetic codon-optimized genes (purchased from GENEWIZ Europe) into the protein over-expression plasmid pET-16b [24] (Novagen), which was then transformed into *E. coli* BL21(DE3) pLysS strain (Novagen). Ten liter cultures were grown at 37 °C in LB medium supplemented with 100 μg/mL ampicillin until $OD_{600}=0.5$, induced with a final concentration of 1 mM IPTG and further grown overnight at 25 °C. Cells were harvested by centrifugation at 17.000× *g* for 30 min, washed in isotonic solution (0.9% NaCl) and resuspended in 400 mL of 50 mM NaH_2_PO_4_, 300 mM NaCl, pH 8 buffer. Cells were then lysed by five freeze-thaw cycles in liquid nitrogen (1 min) and at 50 °C (3 min), and homogenized by sonication (maximum power for 15 min at 50% duty cycle). The soluble fraction was recovered by centrifugation at 12,000× *g* and 4 °C for 60 min. It was then heated to 75 °C for 1 h to remove the non-thermostable proteins from the *E. coli* expression host. Protein debris were removed by centrifugation at 12,000× *g* and 4 °C for 60 min. The extraction was repeated a second time for maximum recovery. The supernatant was concentrated to ∼20 mL by ammonium sulfate precipitation and further purified by Size Exclusion Chromatography on an AKTA^®^ FPLC system, using an XK50-60 column packed with 1 L of Superdex^®^ 75 Prep-Grade resin, calibrated with the GE Healthcare^®^ Low Molecular Weight kit (Figure S2). During this step, both proteins evidenced a dimeric quaternary structure, as they eluted at double the expected MW (Figure S3). Fractions containing the protein were then pooled, concentrated by ultrafiltration (Amicon^®^ Ultra-15 centrifugal filter units, Millipore, Burlington, MA, USA) and lyophilised. The purity of the proteins was assessed by SDS-PAGE, and was greater than 99% (Figure S4). To prepare the samples, the lyophilised protein powder was gently dissolved in D_2_O (Sigma-Aldrich, Saint Louis, MO, USA) under nitrogen atmosphere, at a concentration of 120 mg/mL. Protein solutions rather than hydrated powders were employed in order to optimally transmit hydrostatic pressure to the sample. Proteins employed in the two different experiments belonged to the same production batch. Proteins for crystallization were obtained by employing the same protocol with two modifications: the expression plasmid was pT7-7 [25], which also exploits the T7 expression system but lacks the His-Tag sequence, and the culture volume was scaled down to 1.5 L.

### 4.2. X-ray Crystallography

The purified proteins were diluted to 10 mg/mL in 10 mM Tris-HCl pH 8, 300 mM NaCl. Crystallization trials were performed at 20 °C using the hanging-drop vapour-diffusion method in 96-well micro-plates and a Mosquito HTS robot (TPLabtech) with 100 nL of protein mixed with 100 nL of reservoir. Tko PMI crystals were obtained after one week from condition E8 of the Structure screen 1 + 2 kit (Molecular Dimensions), containing 0.2 M ammonium phosphate monobasic, 0.1 M Tris pH 8.5, and 50% *v/v* MPD. Tba PMI crystals were obtained after two weeks from condition F7 of the Morpheus kit (Molecular Dimensions), containing a 0.12 M monosaccharides mix, 0.1 M buffer system 2 pH 7.5, 30% *v/v* precipitant mix 3. Crystals were flash-frozen in liquid nitrogen. X-ray diffraction datasets were collected at the European Synchrotron Radiation Facility (ESRF, Grenoble) at the ID30A-1 beam line (Massif1 [47,48]) using a pixel detector (PILATUS3 2M) and auto-processed by the XDSAPP package [49]. Tba PMI crystals belong to the I_222_ space group and contain one molecule in the asymmetric unit, while Tko PMI crystals belong to the P_31_ space group and contain eight molecules in the asymmetric unit. The structures of both proteins were determined by molecular replacement with Phaser [50] from the Phenix package [51], using models from the Alphafold2 server [52]. After model building using Coot [53] and Refine [54] from the Phenix package (Tba PMI) or REFMAC5 [55] (Tko PMI), the final structures exhibited an R(%)/R(%)_free_ of 0.18/0.20 at 1.7 Å (Tba PMI) and 0.23/0.29 at 2.2 Å (Tko PMI). Final refinement statistics for the structures are listed in Table S1 and Table S2. The atomic coordinates and structure factors of Tba PMI and Tko PMI have been deposited in the Protein Data Bank with accession numbers 7ZVM and 7ZVY, respectively.

### 4.3. Elastic Incoherent Neutron Scattering (EINS)

EINS measurements were performed on the IN13 backscattering spectrometer at the Institut Laue-Langevin (ILL, Grenoble, France). At the elastic position, IN13 has an incident wavelength of 2.23 Å and a nearly *q*-independent resolution of 8 μeV FWHM, which gives a time window of ∼100 ps [56], allowing for probing local motions of hydrogen atoms since their incoherent scattering cross section is an order of magnitude larger than that of other isotopes [29]. Temperature was controlled by means of a closed-cycle dry cryofurnace (Displex+), and continuous up-scans were performed in the 283 K to 363 K range at 0.08 K/min. The scattering intensity was also measured while the temperature was lowered back to 283 K before the next pressure point to check for hysteresis and, once its absence was verified, the downscans were merged with the upscans to improve statistics. HHP was transmitted to the sample by means of the high-pressure stick, cell and controller developed by the SANE team at ILL [57], and four pressure points were investigated (1, 150, 300 and 600 bar). The high-pressure cell is cylindrical and made of a high-tensile aluminium alloy (7026) and has a 6 mm internal diameter [58]. A piston separates the pressure-transmitting liquid (Fluorinert^™^ FC-770 [59]) from the sample, and a cylindrical aluminium insert (4 mm diameter) was used to decrease sample volume and to minimize multiple scattering. Raw data were corrected for transmission, empty cell and D_2_O scattering, normalized to a vanadium standard and then binned in temperature in 10 K intervals using the LAMP [60] software available at ILL. EINS data have been interpreted in the framework of the *two-state model* [31], which models hydrogen atoms’ motions as a combination of vibrations in two harmonic potential wells, which give the Debye–Waller contribution with the associated Mean Square Displacement (MSD) $\Delta x_{0}^{2}$, and jumps between them. The wells are separated by a distance *d* and have a free-energy difference ΔG that can be separated into the enthalpic and enthropic contributions according to ΔG=ΔH−TΔS. The elastic scattering function S(q,ω=0) as a function of the scattering vector *q* (related to the scattering angle θ and the neutron’s wavelength λ according to $q=\frac{4\pi }{\lambda }sin\left(\frac{\theta }{2}\right)$) thus reads:

(1) $$S\left(q,0\right)=e^{-\Delta x_{0}^{2}q^{2}}\left[1-2p_{1}p_{2}\left(1-\frac{\sin(qd)}{qd}\right)\right],$$

where $p_{1}$ and $p_{2}$ represent the population of each well, assumed in our case to follow the Arrhenius law ($p_{1}/p_{2}=exp\left(-\Delta H/RT+\Delta S/R\right)$, where *R* is the gas constant). It must be stressed, however, that, in the investigated temperature range, large-scale motions could enter the experimental window. It is thus desirable to view the two wells as an average representation of the protein’s free-energy landscape that is accessible at each temperature and pressure value.

### 4.4. Quasi-Elastic Neutron Scattering (QENS)

QENS measurements were carried out on the IN5 time-of-flight (TOF) spectrometer [61] at ILL at 5 Å incident wavelength. In this configuration, the energy resolution was ∼70 μeV HWHM, giving a time window of ∼10 ps, suitable for investigating fast localized protein motions. Temperature was controlled with the standard ILL Orange Cryofurnace in the same range as the EINS experiment, and continuous scans at 0.4 K/min were acquired. The same HHP equipment was used for pressure transmission at 1, 150 and 300 bar. The 600 bar point could not be measured because of time constraints. The same corrections as in the EINS data treatment were applied (see Supplementary Materials) and, after temperature binning, TOF data were further corrected for detector efficiency and detailed balance [28], then converted to S(q,ω) (where ℏω is the energy that a neutron exchanges with the sample) and re-binned in 20 spectra with evenly spaced (0.02 meV) energy points at *q* values from 0.07 to 2.57 Å^−1^. Only spectra having a sufficient dynamic range (−1.5 to +1.5 meV) were considered in the analysis, giving a final *q* range of 0.6–1.8 Å^−1^. The whole treatment was performed with LAMP [60]. First, a model-free analysis of the corrected data was performed. This consists of fitting a sum of Lorentzian functions [62] and leaving their parameters free in order to identify the different dynamical contributions to the measured signal, and then analysing the *q* dependence of their HWHM to define a suitable model that properly fits the data (see Supplementary Materials). Two main contributions have been identified in our case (adding a third Lorentzian did not improve the quality of the fit): the broad component displayed a substantially *q*-independent width, thus representing fast localized motions (e.g., methyl group rotations [30]), while the narrow component’s width exhibited a saturation behaviour at high *q*, characteristic of jump–diffusion processes of protein side-chains [63]. Among the different models that have been tested [38,64,65], the Hall and Ross model [38] gave the most satisfactory results (Figure S14). Therefore, the model function has been built by considering an elastic fraction (represented by the *Elastic Incoherent Structure Factor*, or EISF, $A_{0}\left(q\right)$) plus a q-independent Lorentzian, representing the localized motions [28] ($\Gamma_{loc}$), and then convoluted by another Lorentzian, which represents the jump–diffusion process in the Hall–Ross model [38]. This component is characterized by its *q*-dependent HWHM ($\Gamma_{j}\left(q\right)$), which depends on the time between two successive jumps (τ, also named *residence time*) and the average length by which hydrogen atoms jump (*<l>*). The theoretical scattering function S(q,ω) thus reads:

(2) $$S\left(q,\omega \right)=\frac{A_{0}\left(q\right)}{\pi }\frac{\Gamma_{j}\left(q\right)}{\Gamma_{j}{\left(q\right)}^{2}+\omega^{2}}+\frac{1-A_{0}\left(q\right)}{\pi^{2}}\frac{\Gamma_{j}\left(q\right)+\Gamma_{loc}}{{\left(\Gamma_{j}\left(q\right)+\Gamma_{loc}\right)}^{2}+\omega^{2}}$$

with

(3) $$\Gamma_{j}\left(q\right)=\frac{\hbar }{\tau }\left(1-\exp(-\frac{q^{2}{<l>}^{2}}{2})\right).$$

The model function is then convoluted with the resolution function (derived from a measurement of vanadium, as it is a dominant elastic incoherent scatterer, as shown in Supplementary Materials), multiplied by a *q*-dependent scale factor proportional to the Debye–Waller factor [28], and then fitted to the data using a global fitting approach (i.e., by fitting the whole S(q,ω) at once instead of fitting single spectra at different *q* values separately), which gives the parameters $\Gamma_{loc}$, τ and *<l>*. In order to minimize the number of free parameters and to avoid ambiguities in the global fitting procedure, $A_{0}\left(q\right)$ has been calculated by integrating the spectra in the elastic region, and dividing this value by the total integral of the spectra, following its definition [28]. The calculated $A_{0}\left(q\right)$ have then been used as fixed parameters in the global fitting, permitting to fit the whole S(q,ω) with only three free parameters ($\Gamma_{loc}$, τ and *<l>*) and giving solid and consistent results. Global diffusion of the protein was not taken into account as the broadening arising from it would be lower than the resolution of the instrument in this configuration (see Supplementary Materials). To complete the picture, the geometry of these motions has been characterised by analysing the EISF. It has been modelled taking into account methyl rotation ($A_{3-j}$ with $a_{M}=\sqrt{3}R_{M}=1.715$ Å [63]) and restricted jump–diffusion of protein residues ($A_{j}$, from the Hall–Ross model [38]) according to:

(4) $$\begin{array}{c} A_{0}\left(q\right)=p+\left(1-p\right)\left[sA_{j}\left(q\right)+\left(1-s\right)A_{3-j}\left(q\right)\right]\\ \mathrm{with}\\ A_{j}\left(q\right)=j_{0}^{2}\left(\frac{qR}{2}\right)\\ A_{3-j}=\frac{1}{3}\left[1+2j_{0}\left(qa_{M}\right)\right] \end{array}$$

where *p* represents the fraction of immobile H atoms (i.e., slower than the time-scale of the experiment), *s* is the fraction of H atoms experiencing confinement during their jump–diffusion motion, $j_{0}$ is the zeroth-order Bessel function of the first kind and *R* is the confinement radius.

### 4.5. Molecular Dynamics Simulations

Molecular Dynamics (MD) simulations have been performed with NAMD 2.14 [66] on the P2CHPD computing centre of the Université Claude Bernard Lyon 1. The system was prepared using the CHARMM36m force field on the CHARMM-GUI server [67] by building a cubic water box and leaving a 15 Å padding on each side of the protein. The system was neutralized with 150 mM NaCl, resulting in a total content of 19,407 H_2_O molecules, 55 Na^+^ ions and 59 Cl^-^ ions for Tba PMI, and 17,923 H_2_O molecules, 51 Na^+^ ions and 53 Cl^-^ ions for Tko PMI. The system was first minimized for 20,000 steps, then gradually heated to the desired temperature (310 K or 360 K) by reassigning the velocities every step for 31,000 or 36,000 steps (0.01 K/step) in the NVE ensemble, then equilibrated in the NVT ensemble for 300 ps with a Langevin thermostat (2 ps${}^{-1}$ damping coefficient) and a 2 fs time step, and finally left to evolve in the NPT ensemble at the desired pressure (1 or 400 bar) for 10 ns (2 fs time step) by employing the modified Nosé–Hoover Langevin barostat implemented in NAMD [68], with a 200 fs period and a decay time of 100 fs. Periodic boundary conditions were applied, with particle-mesh Ewald long range electrostatics, using a grid spacing of 1 Å along with a sixth order B-spline charge interpolation scheme. A 12 Å cut-off was used for non-bonded interactions, with a smooth switching function starting at 10 Å. Bonds were constrained using the SETTLE algorithm, and coordinates were output every 10 ps. Analysis of the trajectories was carried out on VMD [69] and, after assessing the stability of the system (Figure S7), the last 5 ns were used to calculate the average coordinates of each atom. This clearly does not correspond to a physical state of the system; therefore, the whole trajectory was aligned with the average structure and the frame with the lowest RMSD (calculated using the $C_{\alpha }$ coordinates) was chosen as the most representative physical state of the system. The same process was repeated for every temperature and pressure condition, and the resulting average structures were used for the calculation of cavities using the CastP server [70]. All the images have been generated using UCSF Chimera 1.16 [71].

## Data Availability

Data are available at http://dx.doi.org/10.5291/ILL-DATA.8-04-876 for the IN13 experiment (8-04-876, from 22 August 2023 or upon reasonable request), and at http://dx.doi.org/10.5291/ILL-DATA.8-05-458 for the IN5 experiment (8-05-458, from 21 September 2023 or upon reasonable request). The X-ray structures of Tba PMI and Tko PMI have been deposited to the Protein Data Bank and are available in the under accession codes 7ZVM and 7ZVY, respectively.

## Supplementary Materials

The following supporting information can be downloaded at: https://www.mdpi.com/article/10.3390/ijms23158469/s1. References [72,73,74,75,76,77,78,79,80] are cited in the supplementary materials.
