# Supplementary material for: Unravelling the Adaptation Mechanisms to High Pressure in Proteins
Source: Int J Mol Sci. 2022 Jul 30;23(15):8469. doi: 10.3390/ijms23158469 (PMC9369236; doi:10.3390/ijms23158469)
Supplement: Supplementary file 1 [file ijms-23-08469-s001.zip › ijms-1828749-supplementary.pdf]

Unravelling the mechanisms of adaptation to high pressure in proteins

Antonino Caliò[1], Cécile Dubois[2], Stéphane Fontanay[1], Michael Marek Koza[3], François Hoh[2], Christian Roumestand[2], Philippe Oger[1], Judith Peters[3,4,5*]

[1] *Univ Lyon, UCBL, INSA Lyon, CNRS, MAP UMR5240, 69622 Villeurbanne, France,*

[2] *CBS, INSERM U1054, CNRS UMR 5048, Univ Montpellier, 34090 Montpellier, France,*

[3] *Institut Laue Langevin, Grenoble, France,*

[4] *Univ Grenoble Alpes, CNRS, LiPhy, Grenoble, France,*

[5] *Institut Universitaire de France, Paris, France*

*corresponding author: jpeters@ill.fr

Supplementary Informations

Genes and proteins sequences

The complete genomes of *Thermococcus barophilus* MP and *Thermococcus kodakarensis* KOD1 are available on GenBank under the accession codes CP002372[1] and AP006878[2] respectively. The two genes are identified as TERMP_00744 (Tba PMI) and TK_0503 (Tko PMI). The codon-optimized sequences used in this work to express the two proteins are reported here:

```
Tba PMI (TERMP_00744)
GCTGAACATATGAAAGCCGAAATTAAGGAGTTCATTGACAGGGGAACTTA
TAGGAAAGCCCCATTGTTTGAAGGTGAGCTTCCTGAAGGGAGTTACGCTC
AAATAGTTGAAATTAAACCCAAGCAGACGGTTCCAAAACACTATCATGAA
AAACAATATGAACTATTTTACATAATTAGCGGACAAGCAAAGCTCGGCAT
TGAAGAAAGAGAATATGATGCAAAACCAGGGGACATATTTTTAGTTAAGC
CCAAGACTGTTCATTGGGTTGTCAATAAAAAGGAAGAGCCATTCAGGCTT
TTTGTGATTAAGCTGAACTACTTTGGAGATGATAGCGTTTGGCTTGAGTG
AGGATCCTTCC

Tko PMI (TK_0503)
GGCTGAACATATGAAAGCAGAAATCAAAAATTTAATTGACCGCGGTACAT
ATCGTAAATTACCGTTATTCGAAGGTGAATTGCCTGAAGGTTCATACGCT
CAAATTGTAGAAGTAAAACCTAAGCAGACTGTAAAAAAACACTACCACGA
ACGTCAATACGAACTGTTCTACATTATCTCTGGTGAAGCTCGTCTTGGTA
TTGGTGATACAGAATATCAAGCAAAGCCTGGTGATATTTTTCTAGTTAAA
CCAAAAACCGTACACTGGGTAGTTAATGAAAAAGACGAACCATTCCGTCT
TTTTGTTGTTAAGTTAAATTATCACGGTGATGACTCTGTATGGTTAGAAT
GAGGATCCTTCC
```

The restriction sites for *NdeI* (blue) and *BamHI* (red) have been indicated. The genes were received from GENEWIZ in pUC57-Kan plasmid, digested with *NdeI* and *BamHI*-HF® (New England Biolabs) and then ligated into the Multi Cloning Site of the pET-16b plasmid (digested with the same enzymes) by means of T4 DNA Ligase (Thermo Fisher). All construct propagation was done using NEB-5α® *E. coli* cells (New England Biolabs). Plasmids were then extracted, purified (Macherey-Nagel NucleoSpin® Plasmid kit) and sequenced to verify the constructs, then transformed into the BL21(DE3) pLysS strain for expression. All the listed products have been employed according to the standard protocols issued by the manufacturers.

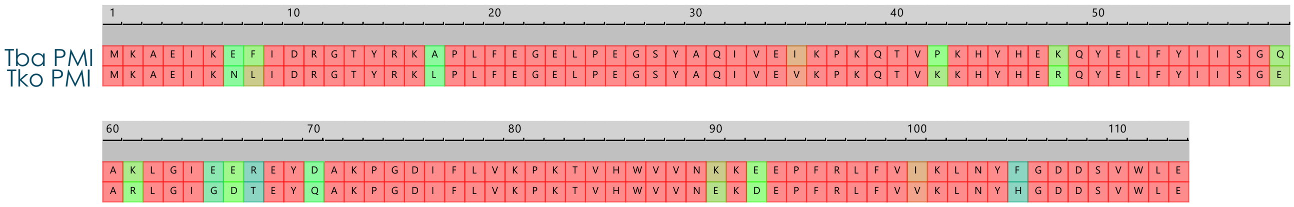

Figure S 1: Sequence alignment of Tba PMI and Tko PMI, color coded for sequence similarity in a Blue-Green-Red scale (blue for least similar, red for most similar).

Figure S1 reports the aligned sequences of the two proteins. They both are 113 residues long, and there are 16 substitutions between them, which are color-coded for sequence similarity (BLOSUM30) in a Blue-Green-Red scale (blue being least similar, red most similar).

Protein production and purification

The proteins have been expressed and purified as reported in the Methods section in the main text. Here we report the calibration run for the XK50-60 column using the GE Healthcare® Low Molecular Weight kit (fig. S2), which consists of five proteins: Conalbumin (75 kDa), Ovalbumin (44 kDa), Carbonic Anhydrase (29 kDa), Ribonuclease A (13.7 kDa) and Aproritin (6.5 kDa). The void volume of the column was determined to be 360 ml by using the Blue Dextran 2000 supplied with the kit. The kit was used according to the manufacturer's protocol.

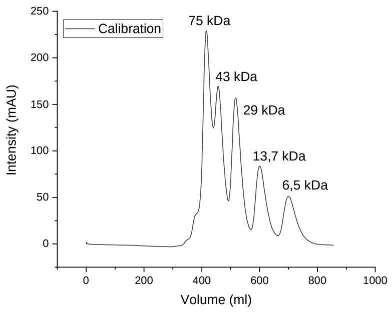

Figure S 2: FPLC calibration run.

Figure S3 reports an example purification run for Tko PMI, showing that it elutes at a volume compatible with a MW of 30 kDa (to be compared with fig. S2), but SDS-PAGE analysis of the fractions shows a band at the 15 kDa mark, thus confirming the dimeric nature of the protein. Fractions from 25 to 48 were pooled together and used as the pure fraction, while the rest of the fractions shown

to contain the protein by SDS-PAGE (fractions 12 to 24 and 49 to 70) were processed separately and kept as a backup.

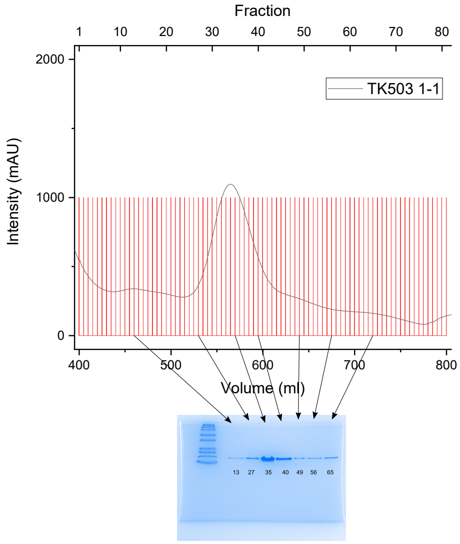

Figure S 3: Example FPLC run. Fractions are given on the upper axis, and the SDS-PAGE analysis of some representative ones is given.

Figure S4 reports an SDS-PAGE analysis of Tba PMI after pooling of the FPLC fractions and concentration, attesting the purity of the protein. The only steps performed after this check were lyophilization, and then the subsequent dissolution in D_2O immediately before the neutron experiments.

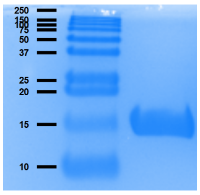

Figure S 4: SDS-PAGE analysis of the final product (Tba PMI).

X-ray Crystallography and Molecular Dynamics

X-ray diffraction datasets were collected at the European Synchrotron Radiation Facility (ESRF, Grenoble) at the ID30A-1 beam line (Massif1[3–9]) using a pixel detector (PILATUS3 2M) and auto-processed by XDSAPP package[10]. The following tables report the final refinement statistics for Tba PMI (Table S1) and for Tko PMI (Table S2).

**Data collection**	**Tba PMI**		
Space group	I_{222}		
Unit cell parameters (a,b,c)	48.500 Å 52.458 Å 112.892 Å		
(α, β, γ)	90.0° 90.0° 90.0°		
Wavelength (Å)	0.96546		
Low resolution limit (Å)	44.562	44.562	1.799
High resolution limit (Å)	1.769	4.799	1.769
Completeness (%)	98.3	96.2	98.6
Multiplicity	5.0	6.0	4.4
I/σ_I	10.2	15.6	4.3
R_{merge}	0.104	0.057	1.015
CC(1/2)	0.994	0.993	0.763
R_{pim}	0.051	0.026	0.533
Total number of observations	71358	4567	3145
Total number unique	14247	763	712
Anomalous completeness (%)	90.9	98.8	82.6
Anomalous multiplicity	2.8	3.6	2.5
**Refinement**			
R_{work}/R_{free} (%)	0.188/0.202		
Number of atoms			
All atoms	1823		
Protein	1758		
Water	64		
Ligands	Mg: 1		
B-factors ($Å^2$)			
Protein	6		
Mg	4		
Water	5		
RMSD			
Bond lengths (Å)	0.0081		
Bond angles (°)	0,97		
Ramachandran favored (%)	97,09		
Ramachandran outliers (%)	0,97		

Table S 1: Refinement parameters for Tba PMI.

Data collection	Tko PMI		
Space group	P_{31}		
Unit cell parameters (a,b,c)	91.16 Å 91.16 Å 113.38 Å		
(α, β, γ)	90.0° 90.0° 120.0°		
Wavelength (Å)	0.96546		
Low resolution limit (Å)	64.79	64.82	2.20
High resolution limit (Å)	2.16	5.87	2.16
Completeness (%)	99.2	99.6	98.4
Multiplicity	2.2	2.2	2.2
I/σ_I	7.8	29.0	0.5
R_{merge}	0.078	0.046	0.661
CC(1/2)	0.977	0.986	0.297
R_{pim}	0.028	0.020	0.628
Total number of observations	387303	6358	33137
Total number unique	40326	869	3632
Anomalous completeness (%)	68.6	79.6	61.8
Anomalous multiplicity	1.3	1.2	1.3
**Refinement**			
R_{work}/R_{free} (%)	0.23/0.29		
Number of atoms			
All atoms	7166		
Protein	7112		
Water	50		
Ligands	Zn: 4		
B-factors (Å^2)			
Protein	68		
Mg	20		
Water	55		
RMSD			
Bond lengths (Å)	0.027		
Bond angles (°)	3.21		
Ramachandran favored (%)	88.24		
Ramachandran outliers (%)	4.2		

Table S 2: Refinement parameters for Tko PMI.

Figure S5 shows the structural alignment of the resulting crystal structures. The substituted residues have also been labelled. Unfortunately, the fast dynamics of the N terminals of both proteins impacted the measured electron densities from the crystals, impeding the characterization of the first two substitutions (E7N and F8L).

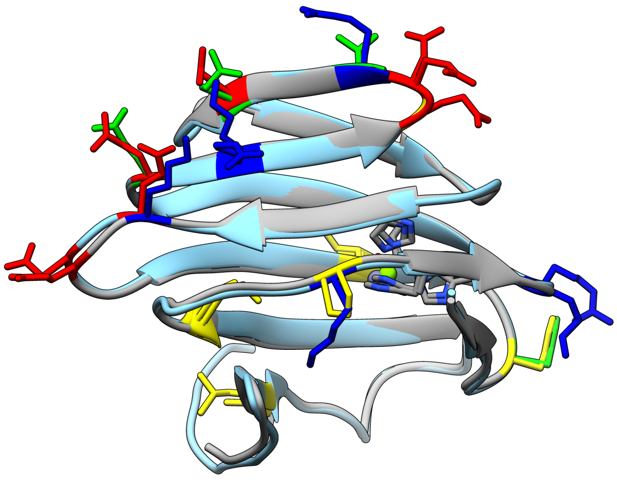

Figure S 5: Structural alignment of the two crystal structures. Tba PMI is shown as gray ribbon and Tko PMI as light blue. Substitutions are shown in sticks and colored by residue type (red for acidic, blue for basic, green for polar and yellow for hydrophobic).

Figure S6 shows the active site, located into the β-barrel and constituted by the conserved residues His44, His46, Glu51 and His85. The ligand is a magnesium ion in Tba PMI and a zinc ion in Tko PMI. However, this assignment is not definitive, as both ions have a Van der Waals radius smaller than the resolution.

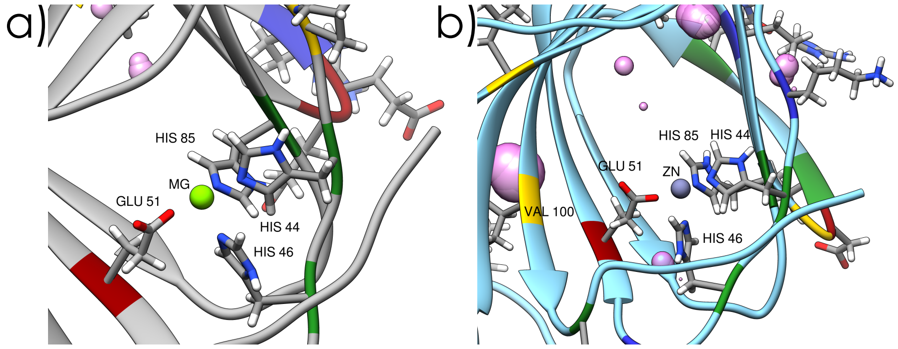

Figure S 6: Detail of the active side of Tba PMI (panel a) and Tko PMI (panel b). The involved residues (His44, His46, Glu51 and His85) and the metal ions (Mg for Tba PMI and Zn for Tko PMI) are shown.

Molecular dynamics simulations were performed as described in the methods section of the main text. Figure S7 shows the C_α RMSD values for the six trajectories.

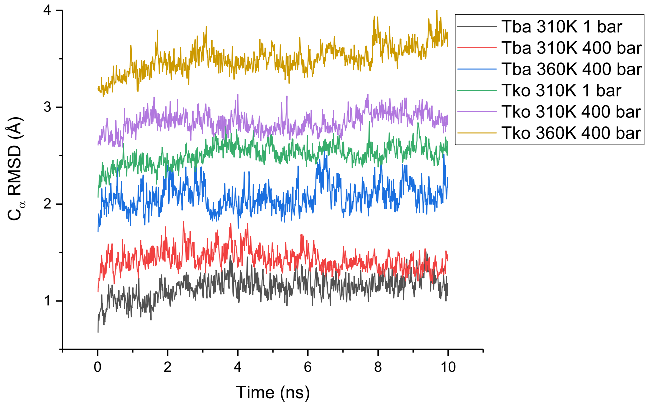

Figure S 7: C_α RMSD values calculated for the six trajectories. Values have been vertically shifted for clarity.

The most representative structures were calculated as described in the main text, and the cavities have been calculated with Castp[11].

Figure S8 shows the surface electrostatic potential of the two proteins (panels a and b) and the vertical section of their ligand pocket. Tba PMI presents a higher positive potential around the mouth of the pocket, and also a higher negative potential in the middle region. This could provide increased shielding from water penetration under high pressure conditions. Moreover, the narrower mouth seemingly plays a role as well in this process.

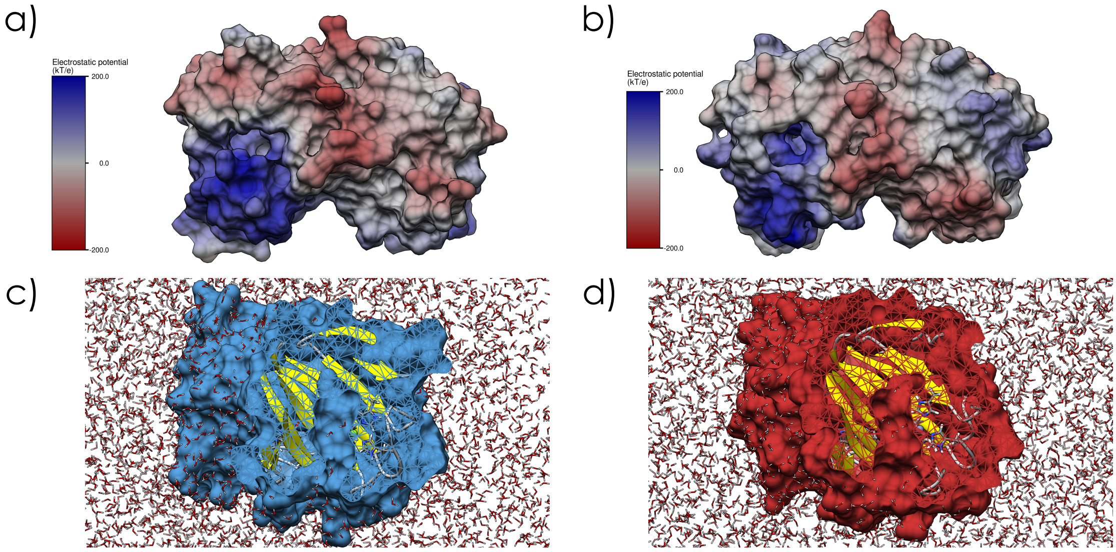

Figure S 8: Surface representation (at 310 K and 1 bar) of Tba PMI and Tko PMI colored by electrostatic potential (panel a and b). One unit of K_BT/e is equivalent to $\sim 27\ mV$ at 310 K. Panel c (Tba PMI) and d (Tko PMI) show a vertical section of the surfaces to highlight the ligand pocket.

Figure S9 shows the equivalent radius calculated in different conditions. After submitting the six equilibrated structures (as described before) to Castp, it returns a value of the molecular volume for each cavity. The two proteins being dimers, the two pockets' volumes were averaged, and then the equivalent radius has been calculated as $R = (\frac{3}{4\pi}V)^{1/3}$, i.e. the radius of a sphere with the same volume. The trend is remarkably similar to that found by QENS.

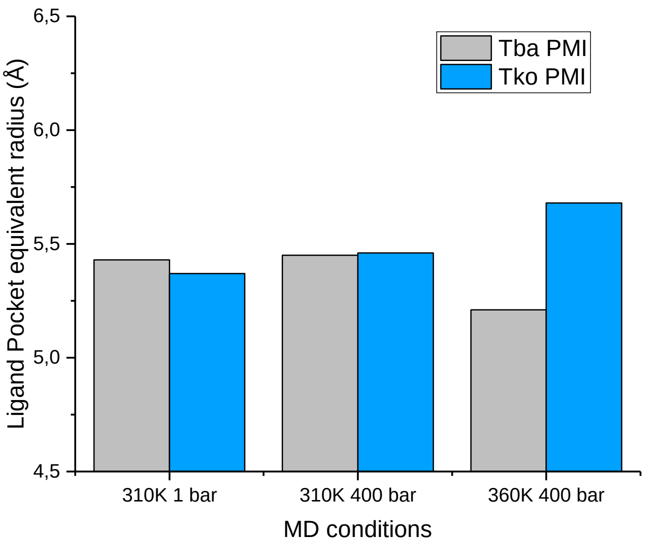

Figure S 9: Equivalent radius of the ligand pocket calculated from the Castp results at different simulation conditions.

Diffusion coefficient calculation

The diffusion coefficient was calculated with HYDROPRO[12] for both proteins at 353 K (at atmospheric pressure). This was the highest temperature (and lowest pressure) reached during experiments, the result would thus give an upper limit for D. Moreover, HYDROPRO calculates D in the infinite dilution limit, therefore we expect the real diffusion coefficient in our samples at 120 mg/ml to be considerably lower. The calculation was performed by using the density and viscosity of pure D_2O[13]. The results were 22.33 $\mathring{A}^2/ns$ and 22.24 $\mathring{A}^2/ns$ for Tba PMI and Tko PMI respectively, thus giving a quasi-elastic broadening of $\Gamma = \hbar D q^2 = 42.5\mu eV$ for Tba PMI, and $42.3\mu eV$ for Tko PMI at the highest q value investigated here. Comparing this with the resolution of IN5 in our experimental conditions (70 μeV HWHM), we can conclude that translational diffusion of the proteins does not give a measurable contribution to the signal.

EINS and QENS Analysis

Both EINS and QENS data were corrected for empty cell, solvent scattering and detector efficiency (measured by means of a vanadium standard) by taking into account their transmission and the solvent volume fraction, according to the following expression:

$$S_{corr}(q,\omega) = \frac{(\frac{1}{t_{sample}}S_{sample} - \frac{1}{t_{cell}}S_{cell}) - \phi(\frac{1}{t_{D2O}}S_{D2O} - \frac{1}{t_{cell}}S_{cell})}{\frac{1}{t_{vana}}S_{vana}} \tag{1}$$

where t denotes the transmission, and the (q,ω) dependence has been dropped for clarity. ϕ denotes the solvent volume fraction in the samples, which has been calculated by dividing the elastic scattering intensity of the solvent by that of the sample at around $q = 1.6\mathring{A}^{-1}$ at each temperature and pressure value, where D_2O displays a broad coherent peak. The transmission can be directly measured on IN13 and the measured values were used for both data sets in virtue of the very weak dependence of the transmission on the neutron wavelength (2.23Å for IN13, 5Å for IN5). The transmission of the samples was 91% for Tba PMI and 93% for Tko PMI, we could thus reasonably assume that multiple scattering effects are negligible. Figure S10 shows the temperature independence of d when fitting the IN13 data with the two-state model[14], thus justifying the global fitting approach with d as a shared parameter.

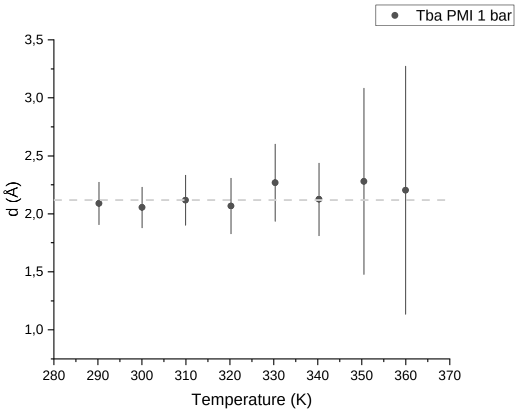

Figure S 10: Temperature independence of the parameter d in the two state model.

Figure S11 displays a vanadium spectrum, from which the resolution function was extracted by fitting a Gaussian curve. Its parameters at all q values were then used to model the resolution function and convolute it to $S(q,\omega)$ to get the experimental scattering function $S_{exp}(q,\omega)$, according to:

$$S_{exp}(q,\omega) = B(q) + \mathcal{R}(q,\omega) \circledast [D(q)S(q,\omega)] \tag{2}$$

where $B(q)$ is a flat background accounting for fast vibrational motions, $\mathcal{R}(q,\omega)$ is the resolution function, $\circledast$ is the convolution operator in ω, $D(q)$ is a scale factor proportional to the Debye-Waller factor and $S(q,\omega)$ is the theoretical scattering function.

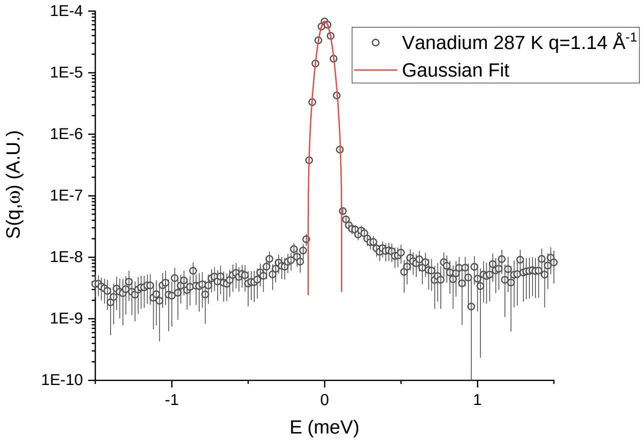

Figure S 11: Spectrum of vanadium at 287 K and at $q = 1.14 Å^{-1}$ (black circles) and gaussian fitting (red line).

We first performed a model-free approach by fitting a sum of Lorentzian functions and leaving their parameters free. As shown in Figure S12, when fitting with three Lorentzians the third always converged to a negligible contribution: its area was two orders of magnitude lower than that of $\mathcal{L}_1$ and $\mathcal{L}_2$, and its width was higher than the instrument's dynamic range in our conditions, therefore two Lorentzians were used. Their HWHM (Γ) are shown in Figure S13 and S14.

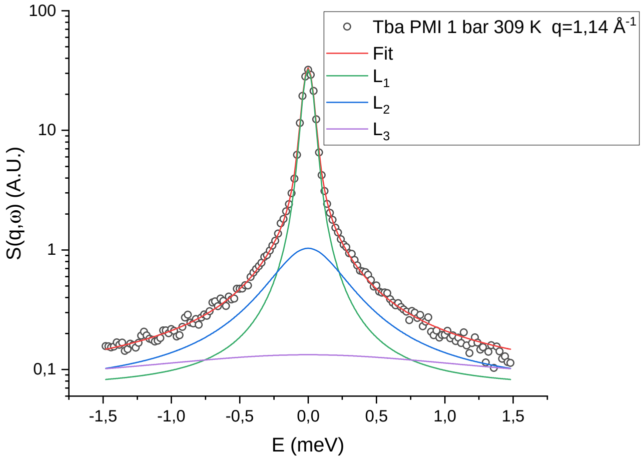

Figure S 12: Example of a three-Lorentzians fitting.

The broad component $\mathcal{L}_2$ shows a rather weak q-dependence (fig. S13), it has thus been assigned to localized motions, which give rise to a q-independent Lorentzian plus an elastic contribution[15],

according to:

$$S_{loc}(q,\omega) = A_0(q)\delta(\omega) + \frac{1-A_0(q)}{\pi}\frac{\Gamma_{loc}}{\Gamma_{loc}^2+\omega^2} \tag{3}$$

where $A_0(q)$ is the *Elastic Incoherent Structure Factor*, and Γ_{loc} is the q-independent HWHM related to localized motions.

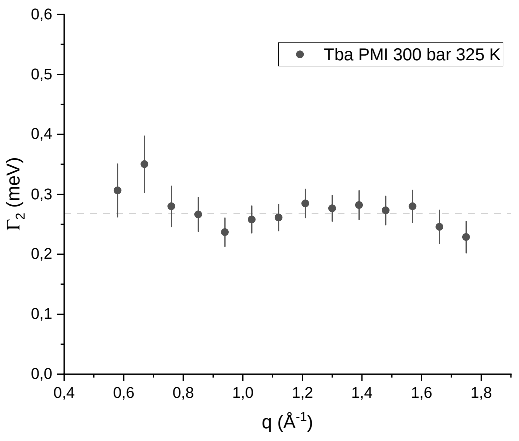

Figure S 13: Width of the broad component as a function of the scattering vector q.

The width of the narrow component $\mathcal{L}_1$ exhibited the signature characteristics of jump-diffusion motions, that is, monotonically increasing at low q and reaching a plateau value at high q (fig. S14). Different jump-diffusion models have been tested[16–18], and the one by Hall and Ross[16] was found to better fit the data compared to the one by Singwi and Sjölander, commonly used to model jump-diffusion motions in proteins.

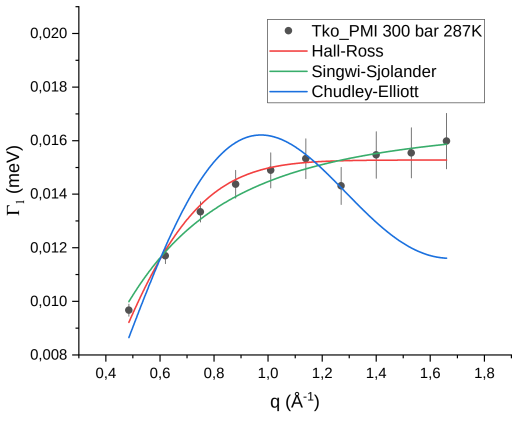

Figure S 14: Width of the narrow component as a function of the scattering vector q.

These motions thus bring about another Lorentzian contribution, according to:

$$S_{jump}(q,\omega) = \frac{1}{\pi}\frac{\Gamma_j(q)}{\Gamma_j^2(q)+\omega^2} \tag{4}$$

with

$$\Gamma_j(q) = \frac{\hbar}{\tau}\left(1-\exp\left(-\frac{q^2\langle l\rangle^2}{2}\right)\right). \tag{5}$$

where τ is the mean time between two jumps (residence time) and $\langle l\rangle$ is the mean jump length, that

is, the mean of the jump distribution function, which is assumed to be Gaussian according to the Hall-Ross model. The overall theoretical scattering function (eq. 2 in the main text) results from the convolution of these two contributions. Since hydrogen atoms motions dominate the QENS signal, a convolution is necessary instead of a simple sum, as they can concomitantly perform both motions. As an example, a methyl group in an isoleucine can rotate (giving rise to S_{loc}) and, at the same time, perform jump-diffusion with the rest of the residue's side-chain (giving rise to S_{jump}).

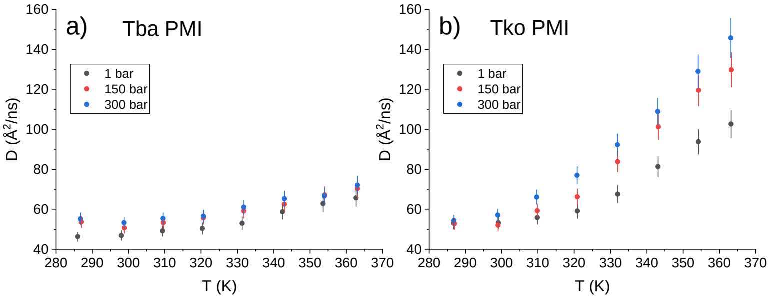

Figure S 15: Pseudo-diffusion coefficient calculated for Tba PMI and Tko PMI at all temperature and pressure values.

Figure S15 shows the pseudo-diffusion coefficient D_{pseudo} described in the main text, which can be used to compare these results with other works.

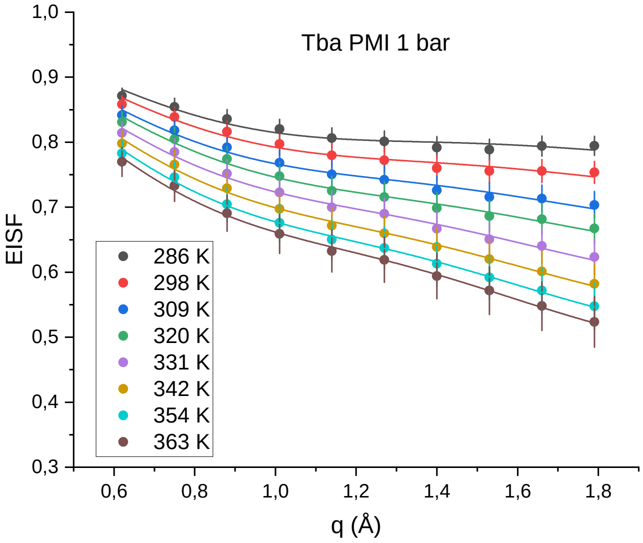

Figure S 16: Example fit of the EISF at all temperatures for Tba PMI at 1 bar.

Figure S16 shows a fit of the EISF at all temperatures for Tba PMI at 1 bar, according to the model defined in equation 4 in the main text, and figure S17 displays the fitting parameters not shown in the main text.

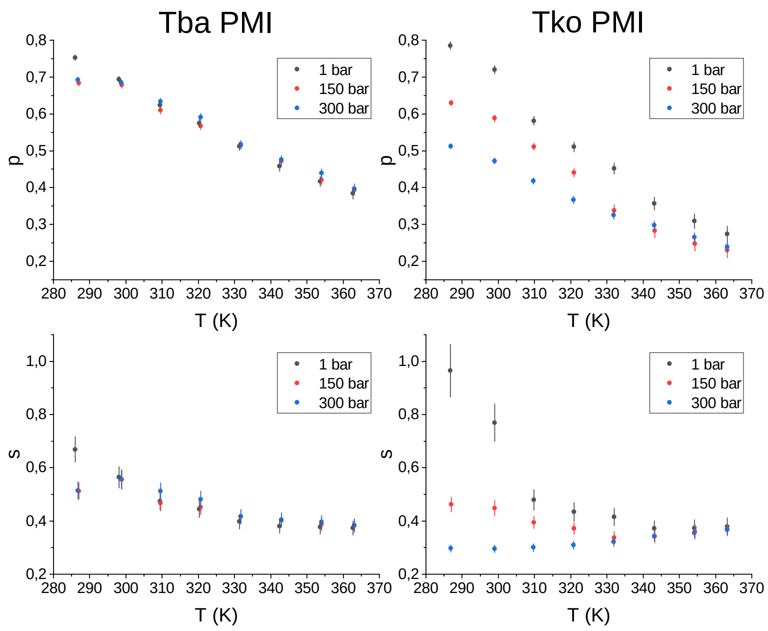

Figure S 17: Temperature independence of the parameter d in the two state model.

Both p (immobile fraction) and s (fraction of atoms performing confined jump-diffusion) are essentially pressure-independent, in line with all the other results, while for Tko PMI it is evident that pressure is activating new motions on one hand (decrease in p), and inhibiting the jump-diffusion motions on the other hand.

References

[1] Vannier, P., Marteinsson, V. T., Fridjonsson, O. H., Oger, P. & Jebbar, M. Complete genome sequence of the hyperthermophilic, piezophilic, heterotrophic, and carboxydotrophic archaeon thermococcus barophilus mp. *Journal of Bacteriology* **193**, 1481–1482 (2011).

[2] Fukui, T. *et al.* Complete genome sequence of the hyperthermophilic archaeon thermococcus kodakaraensis kod1 and comparison with pyrococcus genomes. *Genome research* **15**, 352–363 (2005).

[3] Svensson, O., Gilski, M., Nurizzo, D. & Bowler, M. W. Multi-position data collection and dynamic beam sizing: recent improvements to the automatic data-collection algorithms on MASSIF-1. *Acta Crystallographica Section D* **74**, 433–440 (2018).

[4] Svensson, O., Malbet-Monaco, S., Popov, A., Nurizzo, D. & Bowler, M. W. Fully automatic characterization and data collection from crystals of biological macromolecules. *Acta Crystallographica Section D* **71**, 1757–1767 (2015).

[5] Svensson, O., Gilski, M., Nurizzo, D. & Bowler, M. W. A comparative anatomy of protein crystals: lessons from the automatic processing of 56000 samples. *IUCrJ* **6**, 822–831 (2019).

[6] Bowler, M. W. *et al.* MASSIF-1: a beamline dedicated to the fully automatic characterization and data collection from crystals of biological macromolecules. *Journal of Synchrotron Radiation* **22**, 1540–1547 (2015).

[7] Bowler, M. W., Svensson, O. & Nurizzo, D. Fully automatic macromolecular crystallography: the impact of massif-1 on the optimum acquisition and quality of data. *Crystallography Reviews* **22**, 233–249 (2016).

[8] Nurizzo, D. *et al.* RoboDiff: combining a sample changer and goniometer for highly automated macromolecular crystallography experiments. *Acta Crystallographica Section D* **72**, 966–975 (2016).

[9] Hutin, S. *et al.* Fully autonomous characterization and data collection from crystals of biological macromolecules. *JoVE* e59032 (2019).

[10] Kabsch, W. *XDS*. *Acta Crystallographica Section D* **66**, 125–132 (2010).

[11] Tian, W., Chen, C., Lei, X., Zhao, J. & Liang, J. CASTp 3.0: computed atlas of surface topography of proteins. *Nucleic Acids Research* **46**, W363–W367 (2018).

[12] Ortega, A., Amorós, D. & García de la Torre, J. Prediction of hydrodynamic and other solution properties of rigid proteins from atomic- and residue-level models. *Biophysical Journal* **101**, 892–898 (2011).

[13] Millero, F. J., Dexter, R. & Hoff, E. Density and viscosity of deuterium oxide solutions from 5-70.deg. *Journal of Chemical & Engineering Data* **16**, 85–87 (1971).

[14] Doster, W., Cusack, S. & Petry, W. Dynamical transition of myoglobin revealed by inelastic neutron scattering. *Nature* **337**, 754–756 (1989).

[15] Bée, M. *Quasielastic Neutron Scattering: Principles and Applications in Solid State, Chemistry, Biology and Materials Science* (Adam Hilger, 1988).

[16] Hall, P. L. & Ross, D. Incoherent neutron scattering functions for random jump diffusion in bounded and infinite media. *Molecular Physics* **42**, 673–682 (1981).

[17] Singwi, K. S. & Sjölander, A. Diffusive motions in water and cold neutron scattering. *Phys. Rev.* **119**, 863–871 (1960).

[18] Chudley, C. T. & Elliott, R. J. Neutron scattering from a liquid on a jump diffusion model. *Proceedings of the Physical Society* **77**, 353–361 (1961).
